# Pigmented Villonodular Synovitis (PVNS) of the Knee mimicking Septic Arthritis in a Paediatric Patient: A Case Report

**DOI:** 10.5704/MOJ.2111.019

**Published:** 2021-11

**Authors:** F Indra, IM Anuar-Ramdhan, E Vick-Duin, DN Awang-Ojep

**Affiliations:** 1 Department of Orthopaedics, Universiti Malaysia Sarawak, Kota Samarahan, Malaysia; 2 Department of Orthopaedics, Universiti Malaysia Sabah, Kota Kinabalu, Malaysia; 3 Department of Pathology, Universiti Malaysia Sarawak, Kota Samarahan, Malaysia

**Keywords:** pigmented villonodular synovitis, paediatric knee, septic arthritis

## Abstract

Pigmented villonodular synovitis (PVNS) is a benign but rare proliferative disorder of the synovium. It commonly occurs in the adult population and usually presents as a monoarticular disease. There are two types of PVNS, namely the localised and diffused type. The disease is often misdiagnosed due to its rarity especially in paediatric patients. Knee involvement in PVNS is the commonest form in children although other joints such as hip, foot, ankle, hip, sacroiliac joint and concurrent multiple joint involvements have also been reported. PVNS in paediatric patients is often misdiagnosed as septic arthritis, juvenile rheumatoid arthritis and bone sarcoma, and the diagnosis is usually often made late due to its vague presentation. The majority of PVNS cases are managed by surgery either via open or arthroscopic synovectomy except in a few paediatric patients as described in the literature. This case report of PVNS is of a knee in 11-year-old boy who was initially treated as septic arthritis. The synovium appearance mimicked the features of PVNS during a knee arthrotomy washout, and histopathological examination confirmed the diagnosis. The knee symptoms had significantly improved without additional surgery, and good functional knee motion was achieved, with no sign of recurrence, after two years of follow-up.

## Introduction

Pigmented villonodular synovitis (PVNS) is a benign lesion of the synovial tissues. However, it has the potential to become locally aggressive and cause significant morbidities. It is rarely considered in paediatric patient until multimodality of radiological investigations and tissue studies proved otherwise. The classical presentation of this condition is monoarticular chronic joint effusion but multiple joints involvements have been also reported^1^. Thus, it could mimic other conditions with similar initial presentation, such as juvenile rheumatoid arthritis or septic arthritis^1-4^.

Although it is a benign lesion, the recommended management of PVNS in paediatric age group patients is open or arthroscopic synovectomy^4^. However, surgery can lead to complications such as secondary osteoarthritis, joint instability, joint stiffness and risk of growth plate damage in skeletally immature patients. Furthermore, radiotherapy is also not advisable for paediatric patients to avoid malignant transformation and risk of growth plate injury^4^. We report a paediatric case of knee PVNS, diagnosed and managed as septic arthritis before it turned out to be a PVNS that recovered well without synovectomy.

## Case Report

An 11-year-old boy presented with right knee swelling of three months duration. It was not preceded by trauma. The patient did not have fever or any other joint pain and swelling. He was initially able to walk unaided until one month after the onset before he required crutches to ambulate. He had received symptomatic treatment on seeking medical attention. A month later, he had complained of lethargy and palpitation, and the parent noticed that he appeared pale. He was brought to the tertiary centre and was admitted for further investigation.

On examination, the boy was unable to bear weight on the right leg. He had low-grade fever. He was tachycardic, with normal blood pressure. There were no signs of a haematological disorder such as jaundice, gum bleeding, hepato-splenomegaly or lymphadenopathy. The right knee was warm and tender, with generalised swelling and presence of effusion. A 5x5cm bruise was seen over the medial prepatellar area. The range of motion of the knee was limited to 15° to 45° in both active and passive motion.

Blood investigations showed iron-deficiency anaemia with normal white cell count and platelet level. The C-reactive protein (CRP) and erythrocyte sedimentation rate (ESR) levels were markedly elevated. Other investigations were normal including autoimmune screenings (Anti-nuclear antibodies, C3 and C4 levels) as well as tuberculosis such as sputum cultures and Mantoux test.

**Fig 1:**
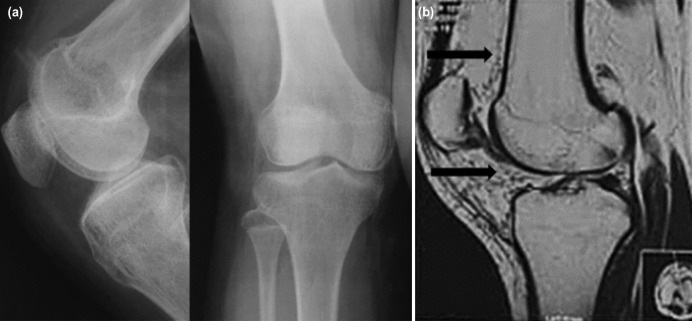
(a) Plain radiograph of the right knee at initial presentation showing no significant bony abnormality or soft tissue swelling. (b) The MRI of the right knee suggests infection, . reported as an aggressive lesion, possibly osteomyelitis of the distal femur with subperiosteal abscess associated with enhancing synovium (black arrow). The mixture of high and low signals within the synovium might represent highly vascularised synovial tissues with low signals hemosiderin deposited tissues.

The right knee radiograph (Fig. 1a) did not reveal any soft tissue swelling or bone infiltration, but knee ultrasonography indicated multiple collections of fluid suggestive of deep-seated infection. The hypoechoic collections were located anterior to the distal femoral shaft, suprapatellar region and medial aspect of the knee. Magnetic resonance imaging (MRI) of the knee suggested aggressive lesion, possibly osteomyelitis of the distal femur, with subperiosteal abscess extending to the proximal tibia, , as well as confirming the suprapatellar pouch collection (as seen in the ultrasonography) with enhanced synovium in the knee, which indicating possible septic arthritis (Fig. 1b).

**Fig 2:**
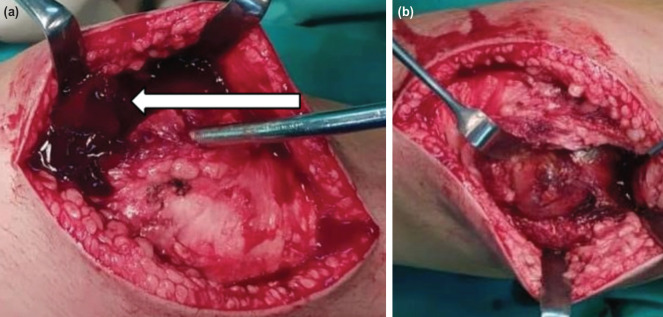
(a) and (b) Intra-operative photographs of right knee with white arrow showing haemarthrosis, and the synovium swollen and reddish.

With the provisional diagnosis of knee septic arthritis, parenteral antibiotics was administered and knee arthrotomy and washout performed using the median parapatellar approach. There was a large hematoma in the joint (Fig. 2a). The synovium appeared to be hypertrophied, reddish and inflamed. Two samples of synovial tissue were biopsied for histopathological examination (Fig. 2b). There was no abscess seen, and the articular surfaces the distal femur and proximal tibia were normal.

**Fig 3:**
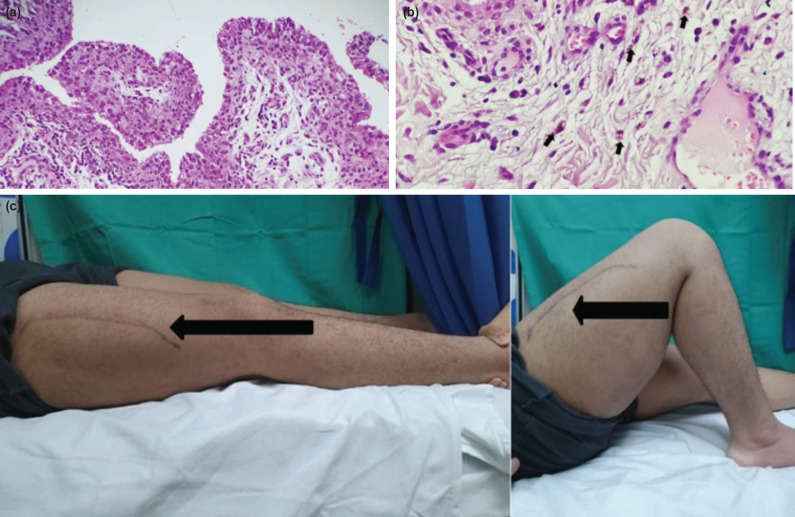
(a) H&E 20x Histopathology examination shows papillary structures lined with the hyperplastic synovial lining, (b) H&E 40x Hemosiderin-laden macrophages (black arrow), (c) Photographs of right knee ROM at two years of follow-up. (The black arrow indicates a surgical scar for femur fracture treatment six months prior to current follow-up).

The intra-articular fluid for culture and sensitivity (C&S) as well as acid-fast bacilli were negative. However, histopathological examination (HPE) of the synovial tissue revealed hyperplastic synovium with short papillary projections (Fig. 3a). The papillary projections were infiltrated with lymphocytes, plasma cells, foamy and hemosiderin-laden histiocytes (Fig. 3b). There was no atypical cell or mitosis seen. These features were compatible with pigmented villonodular synovitis (PVNS).

The patient was initially planned for open synovectomy after the final diagnosis was established. However, the knee pain resolved itself, and the patient was able to ambulate without any aid. Two years later, at follow-up in the clinic, the knee range of motion had markedly improved, from 5° to 110° (Fig. 3c). It was therefore decided to observe his condition. Without any further surgery to remove the synovium, after the two years follow-up, there was no recurrence of symptoms and he was able to resume good daily functional activities.

## Discussion

PVNS is a rare proliferative disorder of the synovial tissues that can occur intra or extra articular. It can present as a localised or diffused type and in single or multiple joints^1^. The diffuse type refers to a generalised involvement of the entire synovial tissues, while the localised type involved part of the synovium or, more commonly, presenting as a pedunculated nodule^2^. It commonly affects the adult population between the age of 20 – 50-years, and rare in the paediatric group^2^, and with no gender preference,. The patient usually presented with joint pain, swelling and stiffness. Although multiple joint involvement have been reported, the knee is the commonest affected joint (80%)^5^. The literature has reported 43 cases of paediatric PVNS, and most of the cases involved the knee joint.

Willimon *et al*^5^ in their paper in 2018 reported a case series of five patients who were eventually diagnosed as hip PVNS. The initial diagnoses made prior to the diagnosis of PVNS were transient synovitis, femoroacetabular impingement, femoral neck stress fracture and bleeding disorders^5^. Juvenile rheumatoid arthritis and bacterial arthritis were the commonest impressions given^5^. Patel *et al* reported a child who presented with left hip pain associated with fever and unable to bear weight. It was presumably treated with antibiotics as a septic hip problem, and the diagnosis of PVNS was made 2½ years later when he came back with a similar complaint^1^. In the present case, the patient was treated for knee septic arthritis before the synovial tissue sampling confirmed the diagnosis of PVNS.

Classically, the joint will show initial clinical signs of swelling or atraumatic hemarthrosis with and limited range of motion. However, in the later stage, joint dysfunction could happen as the cartilage and bone might be heavily infiltrated by the locally aggressive tissues^2,4^. The joint aspiration would exhibit a serosanguinous fluid, and no history of prior trauma could be diagnosed^4^. A brownish or blood-stained fluid from the arthrocentesis had also been previously reported^1^.

In the initial stage of the disease, a plain radiograph might show increased soft tissue density or bony erosion in the advanced stage^1^. Contrast-enhanced computed tomography (CT) scan is useful in assessing the severity of bone loss in a long-standing problem and beneficial to identify recurrent lesions^4^. The best radiographic modality to detect PVNS is MRI. It is extremely useful not only in diagnosing the disease but it can also delineate the extent of the disease for surgical planning^2^. Typically, the MRI will show low signals in both T1 and T2 images due to the deposition of hemosiderin. Some areas with enhanced signals can also be seen due to lipids congestion, effusion and inflamed synovium. Therefore, MRI with contrast is recommended as it will demonstrate generalised enhanced lesions over the synovial tissues as well as bone loss and erosions^1^. However, imaging alone is not pathognomonic. Thus, tissue diagnosis is always necessary to confirm the diagnosis of PVNS, as experienced in this case. As the disease primarily involves the synovial tissues, HPE would demonstrate hypertrophied synovium, the presence of hemosiderin-laden macrophages, histiocytes and osteoclastic giant cells.

The mainstay treatment for symptomatic PVNS is synovectomy. This can be achieved via open surgery or through an arthroscopic procedure. The aims of performing surgery are to prevent further joint destruction, minimise pain and prevent local recurrences^4^. There are controversies on which procedure is considered superior. Some clinicians prefer open surgery as it will enable the surgeons to remove as much as abnormal tissues as possible. Others choose arthroscopic excision as a better option (especially in localised disease) because it produces a similar result with lesser morbidity.

Recently, radiotherapy as the main treatment or in combination with total synovectomy has been used^1^. However, the use of radiotherapy in paediatric cases is still not well accepted. Issues of post-radiation malignant transformation as well as the destruction of the growth plates are the main concern for radiotherapy in children^4^. Nonsurgical management with regular physiotherapy and non-steroidal anti-inflammatory drugs (NSAIDs) for a hip PVNS in a paediatric patient has been described. The patient remained asymptomatic after one year follow-up^1^. Lattanzi *et al* reported the case of an 11-year-old patient with knee PVNS who recovered well within a period of three months and two intra-articular steroids injections. The role of the injections, in this case, had been to delay the surgical management until skeletal maturity was achieved, thus avoiding complications related to growth plate injury during surgery^3^.

The recurrence of the disease is common after surgery. It is frequently due to incomplete excision, especially in the diffused type. In this case, after a two years follow-up, the patient remained active with good functional activities without any synovectomy as the symptoms improved with analgesics and physical therapy.

In conclusion, the diagnosis of PVNS should be considered in a paediatric patient presenting with inability to weight bear and with chronic, insidious joint swelling. An unusual joint problem beyond the classical septic joint presentation must be managed with high index of suspicion and investigated thoroughly with advanced imaging until the correct diagnosis is reached. Non-surgical management of symptomatic PVNS in the knee could be one of the options when dealing with PVNS in a paediatric patient.
